# CHANGES IN LONG-TERM CARE ARRANGEMENTS AMONG OLDER ADULTS WITH ADRD DURING THE FIRST YEAR OF THE COVID-19 PANDEMIC

**DOI:** 10.1093/geroni/igad104.0344

**Published:** 2023-12-21

**Authors:** Emily Wiemers, Yulya Truskinovsky, Amanda Leggett, Geoffrey Hoffman, Vicki Freemdan

**Affiliations:** Syracuse University, Syracuse, New York, United States; Wayne State University, Detroit, Michigan, United States; Wayne State University, Detroit, Michigan, United States; University of Michigan, Ann Arbor, Michigan, United States; University of Michigan, Ann Arbor, Michigan, United States

## Abstract

This study will examine how COVID-19 affected care needs and immediate long-term care (LTC) outcomes including paid and family care, coresidence with adult children, and nursing home use for older adults living with ADRD. For this study we use the 2018 and 2020 waves of the Health and Retirement Study (HRS), a longitudinal, nationally representative survey of individuals aged 51 and older in the U.S. To assess how long-term care arrangements changed among older adults with dementia during the first year of the COVID-19 pandemic, we first describe the needs and LTC outcomes of older adults living with dementia and examine changes between 2018 and 2020 comparing them to changes among older adults with care needs but no dementia. Then, to identify changes associated with COVID-19 (rather than secular trends due to disease progression) we compare the 2020 LTC outcomes of HRS respondents with care needs who had differential exposure to the severity of the pandemic. We proxy pandemic severity with county level COVID-19 deaths. To understand whether changes in LTC outcomes among older adults with dementia were different from the broader population of high-need older adults, we interact local COVID-19 severity with the presence of dementia in 2018. Preliminary findings indicate a retreat from paid care and increases in coresidence with adult children and receiving unpaid help but no significant differences in LTC outcomes among high need older adults with and without dementia.

